# Columbianadin Dampens In Vitro Inflammatory Actions and Inhibits Liver Injury via Inhibition of NF-κB/MAPKs: Impacts on ^∙^OH Radicals and HO-1 Expression

**DOI:** 10.3390/antiox10040553

**Published:** 2021-04-02

**Authors:** Thanasekaran Jayakumar, Shaw-Min Hou, Chao-Chien Chang, Tsorng-Harn Fong, Chih-Wei Hsia, Yen-Jen Chen, Wei-Chieh Huang, Periyakali Saravanabhavan, Manjunath Manubolu, Joen-Rong Sheu, Chih-Hsuan Hsia

**Affiliations:** 1 Graduate Institute of Medical Sciences, College of Medicine, Taipei Medical University, Taipei 110, Taiwan; jayakumar@tmu.edu.tw (T.J.); houshawmin@gmail.com (S.-M.H.); change@seed.net.tw (C.-C.C.); d119106003@tmu.edu.tw (C.-W.H.); m120104004@tmu.edu.tw (Y.-J.C.); m120107013@tmu.edu.tw (W.-C.H.); 2 Department of Cardiovascular Center, Cathay General Hospital, Taipei 106, Taiwan; 3 Division of Cardiovascular Surgery, Department of Surgery, School of Medicine, College of Medicine, Fu Jen Catholic University, New Taipei City 242, Taiwan; 4 Department of Anatomy and Cell Biology, School of Medicine, College of Medicine, Taipei Medical University, Taipei 110, Taiwan; thfong@tmu.edu.tw; 5 Department of Pharmacology, School of Medicine, College of Medicine, Taipei Medical University, Taipei 110, Taiwan; 6 Department of Zoology, Bharathiar University, Coimbatore 641046, Tamil Nadu, India; bhavan@buc.edu.in; 7 Department of Evolution, Ecology and Organismal Biology, Ohio State University, Columbus, OH 43212, USA; manubolu.1@osu.edu; 8 Translational Medicine Center, Shin Kong Wu Ho-Su Memorial Hospital, Taipei 111, Taiwan

**Keywords:** columbianadin, LPS, hydroxyl radicals, HO-1 expression, liver-injury, NF-κB/MAPK signaling pathways

## Abstract

Columbianadin (CBN), a natural coumarin isolated from *Angelica decursiva,* is reported to have numerous biological activities, including anticancer and platelet aggregation inhibiting properties. Here, we investigated CBN’s anti-inflammatory effect in lipopolysaccharide (LPS)-stimulated RAW 264.7 cell activation and deciphered the signaling process, which could be targeted by CBN as part of the mechanisms. Using a mouse model of LPS-induced acute liver inflammation, the CBN effects were examined by distinct histologic methods using trichrome, reticulin, and Weigert’s resorcin fuchsin staining. The result showed that CBN decreased LPS-induced expressions of TNF-α, IL-1β, and iNOS and NO production in RAW 264.7 cells and mouse liver. CBN inhibited LPS-induced ERK and JNK phosphorylation, increased IκBα levels, and inhibited NF-κB p65 phosphorylation and its nuclear translocation. Application of inhibitors for ERK (PD98059) and JNK (SP600125) abolished the LPS-induced effect on NF-κB p65 phosphorylation, which indicated that ERK and JNK signaling pathways were involved in CBN-mediated inhibition of NF-κB activation. Treatment with CBN decreased hydroxyl radical (^•^OH) generation and increased HO-1 expression in RAW 264.7 cells. Furthermore, LPS-induced liver injury, as indicated by elevated serum levels of liver marker enzymes (aspartate aminotransferase (AST) and alanine aminotransferase (ALT)) and histopathological alterations, were reversed by CBN. This work demonstrates the utility of CBN against LPS-induced inflammation, liver injury, and oxidative stress by targeting JNK/ERK and NF-κB signaling pathways.

## 1. Introduction

Inflammation is one of the most common clinical indicators of many disease conditions, including Alzheimer’s, gout, arthritis, and obesity [1]. Therefore, suppression of the inflammatory process is the first significant treatment step in almost all pathological conditions. At present, various chronic inflammatory diseases are controlled by treatment with non-steroidal anti-inflammatory drugs (NSAIDs). However, severe side effects of these treatments are commonly reported [2], necessitating the development of safer anti-inflammatory substances. The molecular mechanisms connected with the inflammatory process are intricate. Among them, the nuclear factor kappa B (NF-κB) and mitogen-activated protein kinase (MAPK) signaling pathways have been found to play significant roles in the inflammatory response. Lipopolysaccharides (LPSs), also known as endotoxins, are found in the outer membrane of Gram-negative bacteria and have been extensively used in models of both systemic and local inflammation. LPS activates macrophage inflammation via activation of NF-κB [3], and controls acute inflammatory and innate immunity functional genes. Rapid phosphorylation of inhibitory κB (IκB) protein results in NF-κB activation by the IκB kinase (IKK) signaling process, which enhances degradation of the IκBα protein [4]. Increased degradation of IκBα subsequently initiates the translocation of active IκB-free NF-κB from the cytoplasm to the nucleus, which results in an activation of promoter regions of target genes and transcription of pro-inflammatory markers, such as tumor necrosis factor (TNF-α), interleukin-6 (IL-6), inducible nitric oxide synthase (iNOS), and cyclooxygenase-2 (COX-2). [5].

NF-κB is closely related to MAPK signaling (the extracellular signaling kinases (ERK1 and 2), the c-jun N-terminal kinases (JNK1-3), and the p38 MAPK (p38α, β, γ, and δ)) pathway, which is responsible for the release of various inflammatory cytokines [6]. In addition, a previous study examined the role of the MAPKs in the induction of iNOS and cytokine expression in activated macrophages [7]. LPS-mediated induction of NF-κB also activates iNOS and inflammatory liver injury [8]. Moreover, the pro-oxidative effect of LPS is generated via the stimulation of reactive oxygen species (ROS) production. ROS act as cellular messengers and provoke an inflammatory response. Due to the imbalance between ROS and antioxidant defense systems, oxidative stress occurs [9]. ROS are reported to induce cell and tissue injury through pro-inflammatory cytokine production and triggering NF-κB [10]. LPS induces iNOS and nitric oxide (NO) at sites of inflammation via activation of p38MAPK and NF-κB in RAW 264.7 cells [11]. Inhibition of JNK has been identified as an important anti-inflammatory mechanism through the suppression of inflammatory genes in several diseases [12]. Evidence shows that inflammation induces ERK activation. Therefore, the reduction of pro-inflammatory mediators and tissue injury via regulating NF-κB/MAPK and free radicals should be effective for treating inflammatory diseases.

Coumarins, the most common secondary metabolites in plants, have numerous physiological activities [13]. Columbianadin (CBN), 1-[(8S)-8, 9-dihydro-2-oxo-2Hfuro [2, 3-h]-1-benzopyran-8-yl]-1-methylethyl-[(2Z)-2-methylbutenoic acid] ester, is a principle component extracted from the root of *Angelica pubescens* Maxim [14]. As an angular dihydrofurocoumarin, it has shown several activities, including antiplatelet activity [15], cytotoxicity against various cancer cells, and in vivo palliative actions in mice [16]. Transportation and absorption kinetics studies revealed that CBN easily entered into the blood and circulated mainly in hepatic tissue [17]. Our recent study also reported the antiplatelet effect of CBN via different molecular mechanisms and suggested that CBN could act as a potential drug to treat thromboembolic disorders [18]. Although several basic biological and anti-inflammatory properties of CBN have been explored, the underlying mechanisms of the anti-inflammatory and liver protective effects are unclear. The present study may provide an improved scientific motivation for its clinical use as a candidate drug for the treatment of inflammatory liver diseases.

## 2. Materials and Methods

### 2.1. Materials

Columbianadin (CBN, >98%) was purchased from ChemFaces Biochem, Wuhan, Hubei, China. Dimethyl sulfoxide (DMSO), PD98059, SP600125, BAY11-7082, and DMPO were purchased from Sigma (St Louis, MO, USA). The antibodies against phospho-p38 MAPK Ser^182^ (pAb), IκBα (44D4), phospho-c-JNK (Thr^183^/Tyr^185^), p38 MAPK, NF-κB p65 (mAb), phospho-p44/p42 ERK (Thr^202^/Tyr^204^), and phospho-NF-κB p65 (Ser^536^) pAb were all purchased from Cell Signaling (Beverly, MA, UAS). Anti-HO-1 pAb was purchased from Enzo (Farmingdale, New York, USA). The monoclonal antibody against α-tubulin was derived from NeoMarkers (Fremont, CA, USA). CBN was dissolved in 0.1% DMSO. All other chemicals and reagents used in this study were commercially purchased from Sigma until specified otherwise.

### 2.2. Cell Cultivation and MTT Assay for Cell Viability

RAW 264.7 cells were procured from the American Type Culture Collection (ATCC, Manassas, VA, USA, TIB-71) and cultivated in DMEM at 37 °C under 5% CO_2_ and 95% air. Cells (2 × 10^5^ cells/well) were pretreated with CBN (10–180 μM) for 20 min, followed by stimulation with LPS (1 μg/mL) for 24 h. An MTT assay was utilized for cell viability. Concisely, a 5 mg/mL MTT working solution was added into each well. After 4 h incubation at 37 °C, the culture medium was collected and 300 µL of DMSO were added to dissolve the crystals. The cell viability index was measured by calculating the absorbance of treated cells/absorbance of control cells ×100%.

### 2.3. Detection of Hydroxyl Radicals

According to our previous study, electron spin resonance (ESR) spectrometry analysis was done [19]. Briefly, RAW 264.7 cells (5 × 10^5^ cells/mL) were exposed to 1 μg/mL LPS after a 20 min incubation with 20 and 40 μM CBN. After a 5 min incubation, the suspensions were added 100 μM DMPO before the ESR analysis was performed. The spectrometer functioned at 20 mW of power, 9.78 GHz of frequency, 100 G of scan range, and 5 × 10^4^ of receiver gain. Variation amplitudes, 1 G; time constant, 164 ms; and scanning for 42 s with 3 scans accumulated.

### 2.4. Immunoblotting Study

For Western blotting analysis, cells and liver tissues were lysed and homogenized using lysis buffer. Fifty micrograms of the extracted proteins were electrophoretically separated using 12% SDS-PAGE and transferred to PVDF membranes and then blocked with 5% skimmed milk. The membranes were subjected to various desired primary antibodies for 2 h. After washing with PBS, they were incubated with HRP-conjugated donkey antirabbit IgG or sheep anti-mouse IgG for 1 h. The immunoreactive bands were identified with an enhanced chemiluminescent (ECL) system. The density of protein bands was quantified by using ImageJ software (NIH, Bethesda, MD, USA). The results were evaluated as relative units determined by normalization of the density of each band to that of the corresponding α-tubulin protein band.

### 2.5. Confocal Microscopy Assay

Cells at a density of 5 × 10^4^ cells per well in 6-well plates on cover slips were pretreated with 40 μM CBN for 20 min followed by stimulation with 1 μg/mL of LPS for 30 min. Cells were fixed with 4% paraformaldehyde for 10 min. After double washing with PBS, cells were permeated with 0.1% Triton X-100 for 10 min and then blocked with 5% BSA for 1 h. Further, the primary p65 antibody was added to cells and kept at 4 °C for overnight. After that, cells were subjected to conjugated goat anti-rabbit IgG for 1 h and then stained with 4,6-diamidino-2 phenylindole (DAPI). Nuclear translocation of NF-κB p65 was examined by using a laser scanning confocal microscope (Nikon Ti-E A1, Tokyo, Japan) and images were captured for further analysis.

### 2.6. Measurement of NO Production

Cells were treated with CBN (20–40 μM) in the presence or absence of LPS (1 μg/mL) for 24 h. Briefly, 100 µL of each culture suspension were incubated with 100 µL Griess reagent for 10 min, and an microplate absorbance (MRX) reader measured the optical density at 550 nm. The NO production was measured with reference to the standard curve of sodium nitrite.

### 2.7. Animals and Experimental Design

Sixty healthy male C57BL/6 mice (weighing 25–30 g) were obtained from BioLasco Taiwan Co., Ld., Taipei, Taiwan. The Institutional Animal Care and Use Committee, Taipei Medical University, Taiwan (LAC-2016-0395) approved the study. After a week of acclimatization in a laboratory condition, the animals were separated into four groups (*n* = 12 per group): (i) Normal saline group (control), (ii) LPS control group (2.5 mg/kg, LPS), and (iii and iv) CBN + LPS groups (10 and 20 mg/kg, respectively). In the drug pretreatment groups, mice were intraperitoneally treated with CBN (10 and 20 mg/kg) for 2 h and then injected with LPS for 6 h. After anesthetizing with isoflurane (induced at 4% and maintained at 3%), blood was collected for biochemical analyses and liver tissues were dissected out for histology study. All mice were in fasting condition prior to collection of blood and tissue.

### 2.8. Histological Analysis

The liver tissues from all animals were fixed in 4% phosphate-buffered formalin for histopathological analysis. The fixed tissues were desiccated and inserted in paraffin and cut into 7 µm thin sections. After overnight drying, sections were dewaxed, rehydrated, and stained using trichrome, reticulin, and Weigert’s resorcin fuchsin. The alterations were observed under a Nikon (Eclipse Ci-L) light microscope (Nikon Co., Tokyo, Japan).

### 2.9. Measurement of Liver Function Enzymes

Markers of liver function, serum aspartate transaminase (AST), and alanine transaminase (ALT) were determined using the Vet-Test^®^ chemistry analyzer (IDEXX, Westbrook, ME, USA) with the results being expressed as international units per liter (U/L).

### 2.10. Statistical Evaluation

The results are given as mean ± standard error (S.E.M). Data were tested using SAS (version 9.2; SAS Inc., Cary, NC, USA). Statistical difference was determined by one-way analysis of variance (ANOVA). If significant variation was identified with multiple comparisons, a Student–Newman–Keuls test was performed. *p* < 0.05 indicated statistical significance. * *p* < 0.05; ** *p* < 0.01; *** *p* < 0.001. The statistical power was calculated with G * Power software (v.3.1) (Heinrich-Heine-Universität Düsseldorf, Düsseldorf, Germany) for animal experimental data. The statistical power was ranged from 0.8–0.9 in all the variables analyzed.

## 3. Results

### 3.1. Effects of CBN on Cytotoxicity, ^•^OH Radical Production, and HO-1 Expression

**Figure 1:**
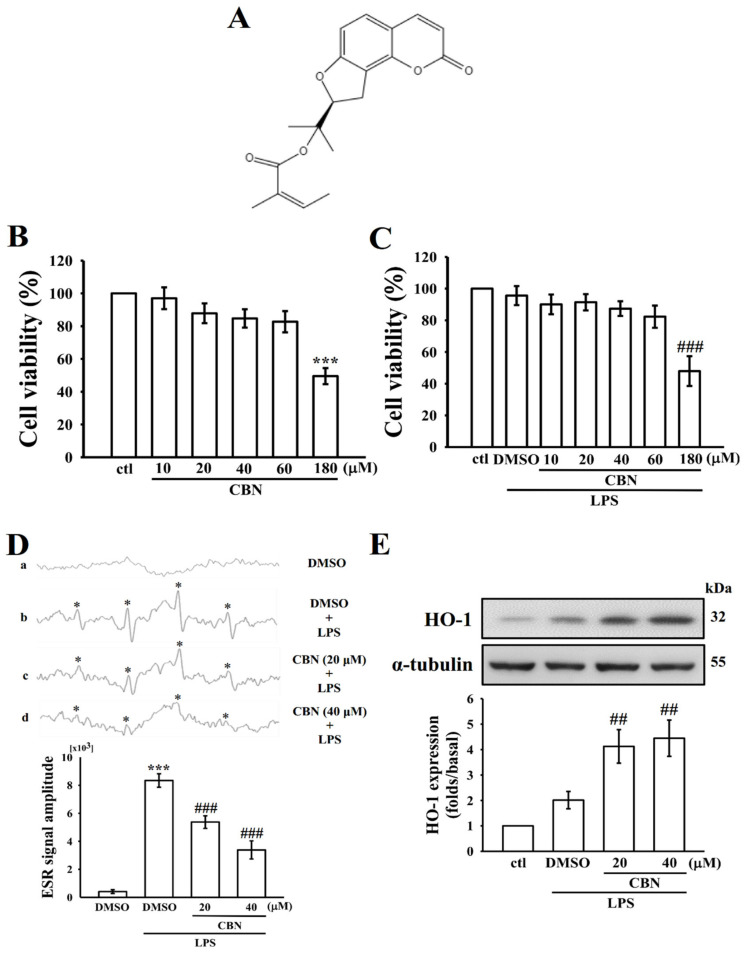
Effect of columbianadin (CBN) on lipopolysaccharide (LPS)-stimulated cytotoxicity, ^•^OH production, and HO-1 expression in RAW 264.7 macrophages. (**A**) The chemical structure of columbianidin (CBN; C_19_H_20_O_5_, molecular weight 328.36). (**B**,**C**) The cell viability of CBN (10–180µM) alone (**B**) or combined with LPS (**C**) as shown in the Materials and Methods section. (**D**) All the reaction mixtures contained (a) 0.1% DMSO, (b) LPS + DMSO, (c and d) CBN (20 and 40 μM) + LPS (1 µg/mL). Radical signal intensity was detected by electron spin resonance (ESR) spectrophotometry. An asterisk (*) indicates the formation of ^•^OH radicals. The statistical significance was calculated from the average values of four identified peaks using WIN-EPR version 921201. (**E**) Cells were treated with 0.1% DMSO or CBN (20–40 μM) for 20 min, and then induced by LPS (1 μg/mL) for 24 h to detect HO-1 expression by immunoblotting. Data presented are the means ± S.E.M. (*n* = 4); *** *p* < 0.001 compared with the control cells; *^##^ p <* 0.01 and ^###^ *p* < 0.001 compared to LPS-induced cells.

To find whether CBN (Figure 1A) produces inhibition of ^•^OH radical production without affecting cell viability, its cytotoxicity was detected by an MTT assay in RAW 264.7 cells. As shown in Figure 1B,C, CBN (10–180 μM) alone or with LPS (1 μg/mL) treatment had no noticeable cytotoxicity up to the concentration of 60 μM. The observed cell viabilities were >90%. Despite CBN showing no toxicity up to 60 μM in RAW cells, we used more safe and effective concentrations of 20 and 40 μM in follow-up experiments. The capability of CBN to scavenge free radicals was measured using the ESR radical scavenging assay and the results are shown in Figure 1D. CBN significantly (*** *p* < 0.001) and concentration (20 and 40 µM) dependently scavenges ^•^OH radicals induced by LPS. Heme oxygenase-1 (HO-1) has been reported as an essential molecular target for anti-inflammatory activity [20] and several natural products have been found to exhibit anti-inflammatory activity via HO-1-mediated NF-E2-related factor 2 (Nrf2) activation [21]. Accordingly, this study tested the effect of CBN on HO-1 protein expression. As shown in Figure 1E, CBN treatment increased HO-1 expression in a concentration-dependent manner. The results may indicate that CBN scavenges LPS-induced ^•^OH radical formation via at least partially increasing HO-1 expression.

### 3.2. CBN Suppresses LPS-Induced MAPK Activation

**Figure 2:**
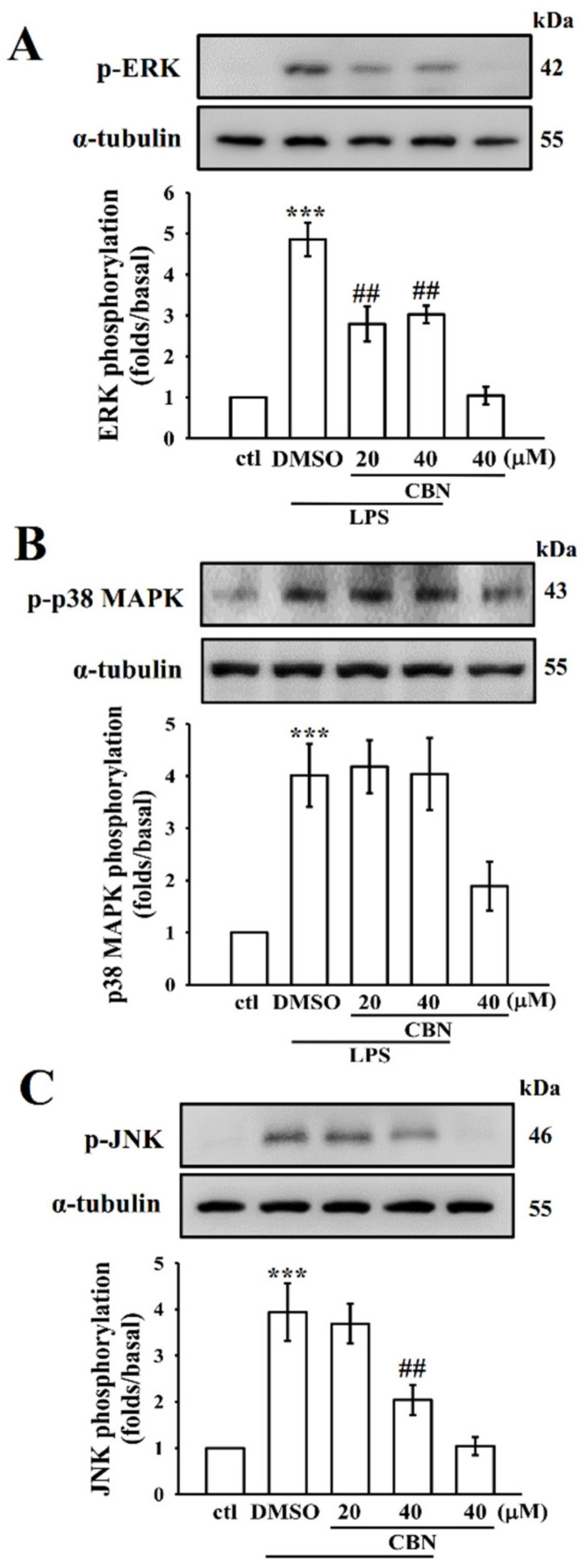
Effects of CBN on ERK1/2, p38MAPK, and JNK1/2 phosphorylation in LPS-induced RAW cells. Cells were treated with 0.1% DMSO or CBN (20–40 μM) for 20 min, and then induced by LPS (1 μg/mL) for 30 min. The (**A**) ERK, (**B**) p38MAPK, and (**C**) JNK phosphorylation was detected by immunoblotting. Data are expressed as the means ± S.E.M. (*n* = 4). *** *p* < 0.001, LPS vs. control cells; ^##^ *p* < 0.01, LPS vs. treatment groups.

Abnormal activation of MAPK signaling results in excessive inflammatory actions, since these molecules are important players in regulating inflammatory-mediated macrophage activation. Inhibition of MAPK activation has been reported to block the activation of inflammatory cytokines and molecules, reducing the severity of inflammatory disease. In this study, as shown in Figure 2, LPS could cause a significant elevation of ERK, p38 MAPK, and JNK phosphorylation after exposure for 30 min. CBN treatment significantly reduced ERK and JNK, but not p38 MAPK phosphorylation. Studies with pharmacological inhibitors demonstrated that MAPK is associated with regulating the transactivation function of NF-κB in macrophages [22]. This result evidenced that ERK and JNK play major roles as regulators of CBN-mediated anti-inflammatory effects in LPS-induced macrophages.

### 3.3. CBN Regulates NF-κB Signaling Pathways

**Figure 3:**
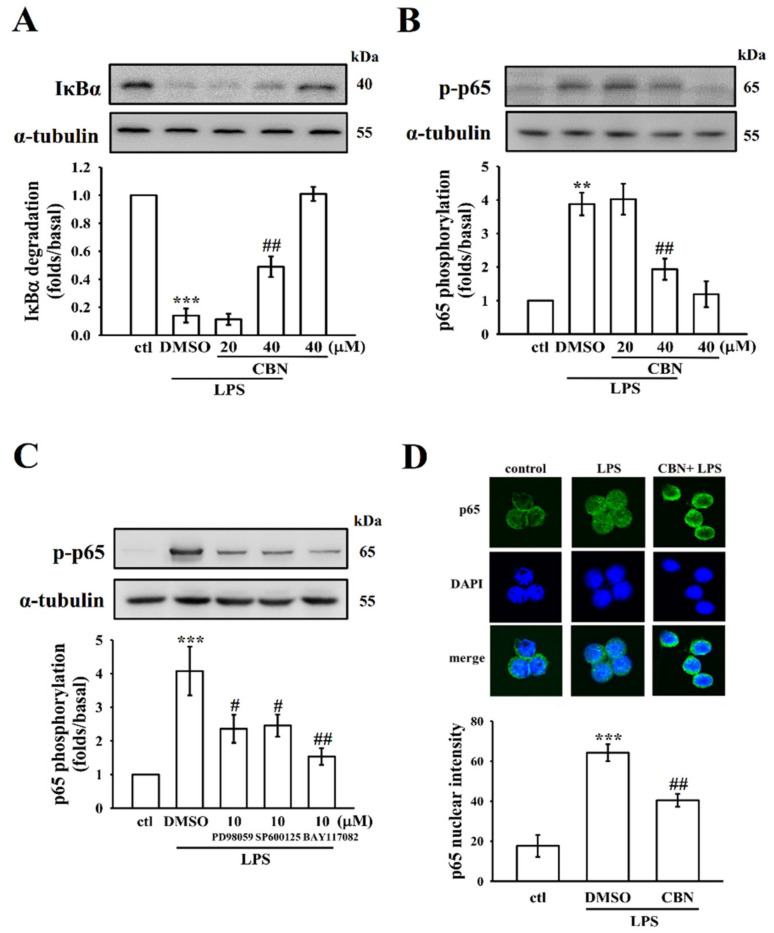
Effects of CBN on LPS-induced NF-κB signaling pathway in RAW cells. RAW cells were treated with CBN (20 and 40 μM) for 20 min with or without LPS (1 μg/mL) for 30 min. Immunoblotting assay was performed to detect (**A**) IκBα degradation and (**B**) p65 phosphorylation. (**C**) Effect of the BAY117082, PD98059, or SP600125 on p65 phosphorylation in LPS-induced RAW cells. (**D**) CBN reversed LPS-induced NF-κB p65 nuclear translocation. Data were graphed by pooling multiple images, with each individual data point corresponding to the mean fluorescence intensity of each individual cell nucleus. ** *p* < 0.01 and *** *p* < 0.001, LPS vs. control cells; ^#^ *p* < 0.05, and ^##^ *p* < 0.01, LPS vs. CBN-treated cells (*n* = 4).

Studies have stated that LPS could activate NF-κB in addition to activating various transcription factors, which regulate numerous inflammatory signals due to its role in stimulating the generation of NO, TNF-α, IL-6, and other inflammatory mediators in activated macrophages [23]. These mediators are also related to the modulation of iNOS. Consequently, in order to investigate the effect of CBN in LPS-induced NF-κB signaling, the levels of IκBα degradation, p-p65 expression, and its nuclear translocation were analyzed. As described in Figure 3A–D, IκBα degradation (Figure 3A), p65 phosphorylation (Figure 3B,C) and its nuclear translocation (Figure 3D) were increased in LPS-induced cells when compared with normal cells. Interestingly, CBN reversed IκBα degradation and inhibited the phosphorylation and nuclear translocation of p65 in LPS-induced cells. Moreover, similar to CBN, specific inhibitors for ERK (PD98059), JNK (SP600125), and NF-κB (BAY117082) significantly reversed LPS-induced p65 phosphorylation (Figure 3C). These results suggest that ERK and JNK1/2 are involved in the upstream signal pathways, which mediate LPS-induced NF-κB activation. Overall, these findings indicate the crucial role of ERK and JNK, but not p38 MAPK, in the upstream regulators of NF-κB activation and play a vital role in the CBN-mediated anti-inflammatory effects.

### 3.4. CBN Reduced the Expression of Inflammatory Mediators

#### 3.4.1. Effects on NO and iNOS

**Figure 4:**
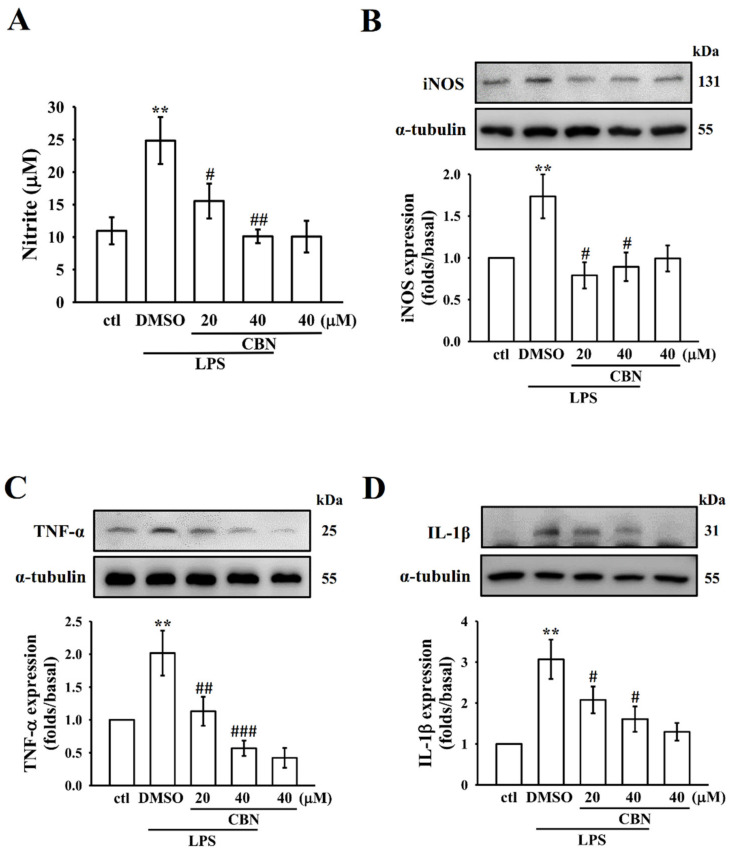
CBN inhibits NO production and expression of iNOS, TNF-α, and IL-1β in LPS-induced RAW cells. The content of NO (**A**) and the expression of iNOS (**B**), TNF*-*α (**C**), and IL-1β (**D**) proteins were assessed as defined in the Materials and Methods. Data presented are the means ± S.E.M. (*n* = 4); ** *p* < 0.01, LPS vs. control cells; ^#^ *p* < 0.05, ^##^ *p* < 0.01, and ^###^ *p* < 0.001, LPS vs. CBN-treated cells.

Elevated levels of NO are considered to be an index for inflammatory disorders and a suitable target to find potent anti-inflammatory agents [24]. The increased production of NO can be attributed to the overexpression of iNOS, which is an important inflammatory mechanism. The NO production and iNOS protein expression were effectively augmented in LPS-induced RAW 264.7 cells (Figure 4A,B), however, co-treatment with CBN significantly reduced the extent of augmentation. This result suggests that the inhibition of NO production by CBN may be connected with its suppressive effect on LPS-induced iNOS protein.

#### 3.4.2. Effects on Inflammatory Cytokines

The anti-inflammatory activity of CBN was evaluated by its inhibitory effect against LPS-induced TNF-*α* and IL-1β expression in the RAW 264.7 macrophages. The expression of pro-inflammatory cytokines TNF-*α* and IL-1β was significantly increased in LPS-induced macrophage cells (*p* < 0.001). As shown in Figure 4C,D, CBN treatment significantly inhibited LPS-stimulated inflammatory cytokine expression, with greater inhibition occurring at higher CBN treatment concentrations.

#### 3.4.3. Effects of CBN on Hepatic Histopathology

**Figure 5:**
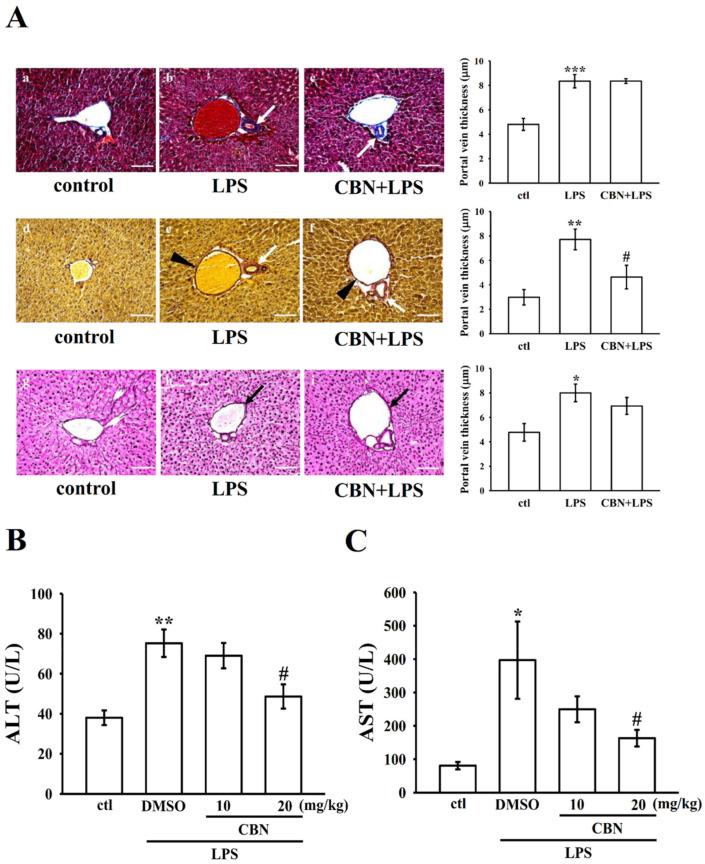
CBN dampens the LPS-induced hepatic injury in mice. (**A**) Effect of CBN on the histological alterations in liver tissue. Trichrome stain (**a**–**c**). The white arrow indicates the collagen deposition in the portal triad region of liver tissues. More abundant collagen fibers surrounded by arteriole and bile duct in LPS- and CBN + LPS-treated groups as compared to the control group. Weigert’s resorcin fuchsin stain (**d**–**f**). Collagen fibers (red fibers indicated by white arrows) and elastic fibers (dark blue fibers indicated by black arrowheads) in control, LPS, and CBN + LPS groups. Reticulum stain (**g**–**i**). A network of fine black reticular fibers identified between the sinusoids and the plates of hepatocytes (indicated by black arrow) in the control, LPS, and CBN + LPS groups. The thickness of the portal vein was calculated using MShot Image Analysis System. Bar = 100 μm. (**B**,**C**) CBN reduces LPS-induced serum alanine aminotransferase (ALT) and aspartate aminotransferase (AST) activities. Illustrative views of control, LPS, CBN (20 mg/kg) + LPS groups are presented (magnification 20x). Data presented are the means ± S.E.M. (*n* = 6); * *p* < 0.05, ** *p* < 0.01, and *** *p* < 0.001, LPS vs. control group; ^#^ *p* < 0.05, LPS vs. CBN group. The statistical power range was from 0.8–0.9.

The production of inflammatory factors in liver tissues is complicated in liver injury. In the histological observation, collagen depositions were profusely distributed in the portal triad regions of liver tissues in LPS-induced mice (Figure 5(Ab)). Thick bundles of collagens were identified as blue and red in trichrome and Weigert’s resorcin fuchsin staining, respectively (indicated by white arrows, Figure 5(Ab,e)). The existence of elastic fibers was also observed in the portal triad region of liver tissues in LPS-induced mice (indicated by black arrowheads, Figure 5(Ae)). As shown in Figure 5(Ab,e,h), the thickness of the portal vein was significantly increased in the LPS group compared with the control group. Remarkably, a high dosage of 20 mg/kg CBN treatment markedly reduced LPS-induced collagens and elastic fibers in liver tissues, as shown in Figure 5(Ac,f). In the reticulum staining, the intralobular stroma appeared as a network of reticular fibers between the sinusoids and the plates of hepatocytes (fine black fibers, Figure 5(Ah)). However, reticular fibers in the LPS-induced liver tissues in mice were more abundant than those in the control and CBN-treated mice (Figure 5(Ag,i)), as indicated by black arrows. These results indicated that administration of CBN could dampen the LPS-induced increment of elastic fibers following injury in liver cells.

### 3.5. CBN Reduces Liver Marker Enzymes ALT and AST

Serum ALT and AST transaminases were determined to examine LPS-induced liver function. A significant increase in activity of ALT and AST in LPS-induced mice compared with normal mice was observed (*p* < 0.05; Figure 5B,C). Treatment of CBN significantly reduced the activities of AST and ALT (*p* <0.05) at a high dose of 20 mg/kg, however, no significant effect was found at 10 mg/kg.

### 3.6. Effects of CBN on Liver MAPKs, NF-κB, Inflammatory Cytokines, and iNOS Protein Expression

**Figure 6:**
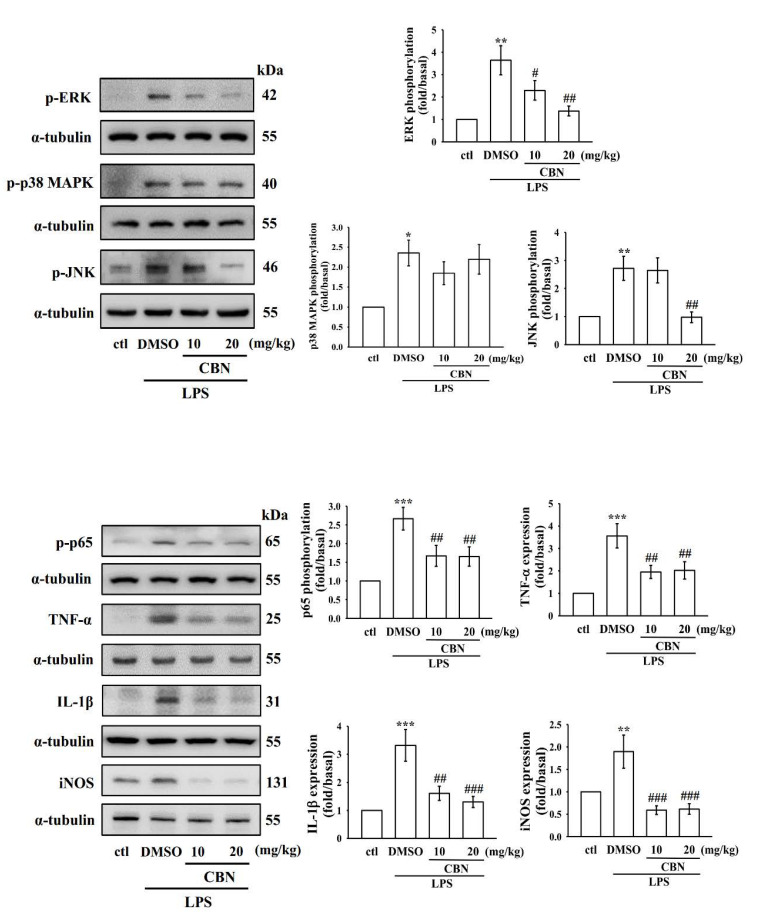
Effects of CBN on the expression of MAPK/NF-κB p65, TNF-α, and IL-1β and iNOS in LPS-induced liver tissue. Mice were treated with CBN (10 and 20 mg/kg) for 2 h and then induced by LPS (1 μg/mL) for 6 h. The expression of phosphorylated ERK, p38, JNK, and p65 and the protein expression of TNF-α, IL-1β, and iNOS were evaluated as described in the Materials and Methods. Data presented are the means ± S.E.M. (*n* = 6); * *p* < 0.05, ** *p* < 0.01, and *** *p* < 0.001, LPS vs. control group; ^#^ *p* < 0.05, ^##^ *p* < 0.01, and *^###^ p* < 0.001, LPS vs. CBN group. The statistical power range was from 0.8–0.9.

The obtained results from the in vitro anti-inflammatory effects of CBN were further substantiated by examining the protein expression of MAPKs, NF-κB, inflammatory cytokines, and iNOS in liver tissues of LPS-induced mice. Similar to RAW cells, proteins were significantly elevated in LPS-exposed mouse liver tissues compared to normal mice (Figure 6). Pretreatment with CBN prominently reduced the expression of the phosphorylation of ERK, JNK, p65, TNF-α, IL-1β, and iNOS. This result indicates that CBN has a hepatoprotective effect via inhibiting these molecules.

## 4. Discussion

Studies have demonstrated that the production of inflammatory cytokines during the stimulation of macrophages plays a vital role in organ damage, including acute and chronic hepatic injury [24]. LPS induces liver injury by controlling oxidative stress and free radicals in hepatocytes [25]. Here, we showed that the ERK/JNK signaling molecules are important in the NF-κB-mediated induction of in vitro inflammatory cytokines, mediators, and in vivo liver injury, and that CBN administration is an effective modulator of inflammatory events. This study showed that free radicals are an important regulator of inflammation and that CBN treatment blocked hydroxyl radical formation and increased HO-1 expression in LPS-induced macrophage cells. These findings indicated that CBN offers its protective effect in LPS-induced macrophage inflammation at least in part through NF-κB- and MAPK-dependent mechanisms.

Reactive oxygen species activate superoxide anion, hydrogen peroxide, and hydrogen radicals. ROS are associated with different cellular and biological functions, such as controlling homeostasis, which is a significant factor for cell growth and survival [26]. However, the excess ROS formation will involve and play a significant role in the majority of pathophysiological events, including inflammation, via initiating intracellular pro-inflammatory mediators [27]. Moreover, ROS enhances MAPK and NF-κB activation in macrophages, resulting in the overexpression of genes, and can lead to the induction of inflammatory events [28]. A previous study demonstrated that ROS plays a dynamic role in septic shock and organ failure [26]. In addition, ROS is elevated in LPS-induced liver injury and antioxidants might be ideal agents to improve recovery from this injury [29]. In this study, we found that LPS significantly elevated the levels of hydroxyl radicals (^•^OH), which are inhibited by CBN in a concentration-dependent manner. Our result is substantiated with the findings of Jiang et al. [30] who found that LPS elevated the levels of free radicals, and that sophocarpine, an alkaloid, suppressed this elevation. Choi et al. reported that Bis (3-bromo-4,5-dihydroxybenzyl) ether, a bromophenol compound derived from a red alga, *Polysiphonia morrowii,* repressed LPS-induced inflammatory mediators by hindering the ROS-mediated ERK signaling pathway in RAW 264.7 cells [31]. Moreover, activation of HO-1 is a common response to oxidative stress as a cellular defense mechanism. Numerous lines of evidence show that expression of HO-1 induced by pharmacological treatment results in the reduction of cellular damage and inflammation. For instance, an anti-inflammatory role has been proposed for HO-1 [32]. This result is consistent with our finding that CBN enhances HO-1 expression in LPS-induced cells. Our results have shown that CBN ameliorates LPS-induced oxidative stress, as indicated by the suppressed ^•^OH production and increased HO-1 expression in RAW cells. These findings indicate that CBN could act as either a ROS inhibitor or antioxidant.

Mitogen-activated protein kinases play an important role in biological signal transduction into the nucleus from the cell membrane. MAPKs play a crucial role in various cellular processes, including gene expression, proliferation, cellular stress, and inflammatory response [33]. Studies have established that LPS could induce phosphorylation of ERK1/2, JNK, and p38 MAPKs in murine macrophages [34]. Further, the suppression of the MAPK pathway has been identified as an effective treatment to reduce inflammation [35]. NF-κB is involved in LPS-induced inflammatory signaling pathways, using inhibitory factor IκB in a resting condition. In LPS-activated cells, the NF-κB p65 subunit is detached due to IκB phosphorylation. Phosphorylated p65 translocates into the nucleus, where it regulates NF-κB-dependent target genes, including inflammatory mediators and cytokines [36]. Milani et al. [37] reported that β-carotene arrests nuclear translocation of NF-ĸB p65, which is correlated with its inhibitory effect on phosphorylation, and degradation of the NF-ĸB inhibitor. Similarly, β-carotene diminishes IκB phosphorylation and significantly hinders the nuclear translocation of NF-κB p65 [38]. Our earlier study revealed that CME-1, a novel polysaccharide, inhibited IκBα degradation and p65 Ser536 and MAPK phosphorylation in LPS-stimulated RAW 264.7 cells [39]. Our data support these results, showing that CBN inhibits NF-κB p65 nuclear translocation via hindering p65 phosphorylation on Ser536 and IκB degradation. Moreover, we used NF-κB (BAY 117082), ERK (PD98059), and JNK (SP600125) inhibitors to further explore the possible involvement of MAPK in NF-κB activation in CBN-mediated anti-inflammatory activity, and showed that CBN inhibits ERK and JNK in macrophage cells. The results revealed that that ERK1/2 and JNK signaling pathways mediate the LPS-induced NF-κB activation, inhibiting p65 phosphorylation. These findings also indicate the crucial role of ERK and JNK, but not p38MAPK, in the upstream regulator of NF-κB activation and they play vital role in the CBN-mediated anti-inflammatory effects.

LPS could induce inflammatory responses via elevating cytokines such as TNF-α, IL-6, and IL-1β, and hence inhibition of these cytokines could diminish the response of inflammation. The increased expression of iNOS can be attributed to the development of inflammation and liver injury, as this elevation can induce the production of NO, which is an additional stimulator involved in oxidative activity that in turn is involved in inflammation [35]. Moreover, NO is involved in a wide range of oxidative reactions, and LPS could induce a substantial increase in NO levels because of the elevated synthesis of iNOS. Hence, NO activation may be an initial marker of liver damage, and inhibition of this molecule could be a target for regulating inflammation [40]. In this study, CBN significantly reduced the expression of TNF-α, IL-1β, and iNOS in RAW cells and liver tissues, which confirmed CBN’s anti-inflammatory and hepatoprotective effects. These results are consistent with an earlier study that reported anti-inflammatory effects of the polyphenol-enriched fraction (PEF) from *Acalypha wilkesiana* against LPS-induced inflammation [41]. It was found that PEF attenuated LPS-induced NO production and suppressed iNOS expression, and it also reduced the secretion of TNF-α, IL-1β, and IL-6 in LPS-stimulated macrophages. Our results also support those of Zhang et al. [42], who found that CBN suppressed TNF-α and IL-1β in the culture supernatant of THP-1 cells that had been stimulated by LPS.

Liver injury induced by LPS is linked with inflammatory mediators including superoxide, nitric oxide, TNF-*α*, IL-1*β*, IL-6, and other cytokines [43]. LPS stimulates NF-*κ*B, leading to the initiation of many inflammatory genes, such as TNF-*α* and IL-1*β* [44]. Therefore, inactivation of NF-*κ*B could attenuate LPS-induced liver injury. Studies have indicated that antioxidant and anti-inflammatory agents are valuable in LPS-induced hepatic injury [45]. This study indicated that administration of LPS caused increased ALT and AST activities in serum, measured as markers of liver injury. LPS-induced tissue injury results from an increase in the release of cytokines and oxidative stress [46], resulting the stimulation of apoptosis of hepatic cells and necrosis [47]. In this study, CBN pretreatment reduced the increases in ALT and AST enzymes and lowered the infiltration of inflammatory cells, fibrosis (elastic and reticular fibers), and collagen deposition induced by LPS in the liver tissues of mice, which were evidenced by trichrome, Weigert’s resorcin fuchsin, and reticulum stains. Consistent with the in vitro findings, CBN also inhibits MAPKs and p65 phosphorylation, cytokines, and iNOS expression in the LPS-induced mouse liver. These results together specified the anti-inflammatory and hepatoprotective effect of CBN and its therapeutic potential for inflammatory diseases.

## 5. Conclusions

CBN exhibits compelling anti-inflammatory and hepatoprotective effects by preventing free radical formation and decreasing the expression of MAPK, especially ERK and JNK, followed by the suppression of NF-κB pathways. This in turn led to inhibition of NO, iNOS, TNF-α, and IL-1β in LPS-activated RAW cells and mouse liver. This study found that ERK and JNK act as upstream mediators of NF-κB activation in LPS-induced cells. CBN shows an antioxidant effect via increasing HO-1 expression in LPS-induced RAW cells. The detailed histological evaluation showed that CBN protects LPS-induced liver fibrosis as evidenced by decreased collagen, elastic, and reticular fibers. Though further study of the principal mechanisms is required, we concluded that CBN may offer a protective mechanism by controlling multiple signaling cascades, and this natural coumarin derivative could be used as a drug candidate for treating inflammation-mediated diseases.

## Data Availability

The data presented in this study are available upon request on case-to-case basis.
